# Severe loss-of-function mutations in the adrenocorticotropin receptor (ACTHR, *MC2R*) can be found in patients diagnosed with salt-losing adrenal hypoplasia

**DOI:** 10.1111/j.1365-2265.2006.02709.x

**Published:** 2007-02-01

**Authors:** Lin Lin, Peter C Hindmarsh, Louise A Metherell, Mahmoud Alzyoud, Maryam Al-Ali, Caroline E Brain, Adrian J L Clark, Mehul T Dattani, John C Achermann

**Affiliations:** * UCL Institute of Child Health & Department of Medicine, University College London London, UK; † Department of Endocrinology, Barts and the London, Queen Mary, University of London West Smithfield, London, UK; ‡ Department of Paediatric Endocrinology, Hamad Medical Centre Doha, Qatar

## Abstract

**Objective:**

Familial glucocorticoid deficiency type I (FGD1) is a rare form of primary adrenal insufficiency resulting from recessive mutations in the ACTH receptor (MC2R, *MC2R*). Individuals with this condition typically present in infancy or childhood with signs and symptoms of cortisol insufficiency, but disturbances in the renin-angiotensin system, aldosterone synthesis or sodium homeostasis are not a well-documented association of FGD1. As ACTH stimulation has been shown to stimulate aldosterone release in normal controls, and other causes of hyponatraemia can occur in children with cortisol deficiency, we investigated whether *MC2R* changes might be identified in children with primary adrenal failure who were being treated for mineralocorticoid insufficiency.

**Design:**

Mutational analysis of *MC2R* by direct sequencing.

**Patients:**

Children (*n* = 22) who had been diagnosed with salt-losing forms of adrenal hypoplasia (19 isolated cases, 3 familial), and who were negative for mutations in DAX1 (NR0B1) and SF1 (NR5A1).

**Results:**

*MC2R* mutations were found in three individuals or kindred (I: homozygous S74I; II: novel compound heterozygous R146H/560delT; III: novel homozygous 579-581delTGT). These changes represent severely disruptive loss-of-function mutations in this G-protein coupled receptor, including the first reported homozygous frameshift mutation. The apparent disturbances in sodium homeostasis were mild, manifest at times of stress (e.g. infection, salt-restriction, heat), and likely resolved with time.

**Conclusions:**

*MC2R* mutations should be considered in children who have primary adrenal failure with apparent mild disturbances in renin-sodium homeostasis. These children may have been misdiagnosed as having salt-losing adrenal hypoplasia. Making this diagnosis has important implications for treatment, counselling and long-term prognosis.

## Introduction

Familial glucocorticoid deficiency type 1 (FGD1) (MIM: 202200) is an autosomal recessive ACTH–resistance syndrome resulting from mutations in the ACTH receptor (melanocortin-2 receptor, *MC2R*[MIM: 607397]).^1^,^2^ The molecular basis of this condition was first described in 1993 and, to date, approximately 20 different MC2R mutations have been reported in around 40 individuals or families with FGD1·^3^–^11^ The majority of MC2R mutations are homozygous or compound heterozygous missense mutations that impair the function of this G-protein coupled receptor to varying degrees.^10^,^11^ To our knowledge, homozygous frameshift or nonsense mutations in MC2R have not been described previously.

Children with FGD1 typically present with symptoms and signs of glucocorticoid deficiency, such as prolonged jaundice, hypoglycaemia or hypoglycaemic convulsions, failure to thrive, hyperpigmentation and sepsis.^1^,^2^ Tall stature has also been reported in a subset of individuals with FGD1, especially prior to glucocorticoid treatment, but the pathogenic basis of this feature is not clear.^6^,^12^ Disturbances in the renin-angiotensin system, mineralocorticoid synthesis or sodium homeostasis are not a well-established feature of this condition, and patients with classic FGD1 do not receive mineralocorticoid replacement. Nevertheless, the MC2R is expressed in the zona glomerulosa of the adrenal gland, and acute administration of ACTH(1–24) results in aldosterone release in normal human volunteers.^13^–^15^ Thus, a facilitative role for ACTH in supporting mineralocorticoid synthesis may be physiologically important, perhaps during stress or in early life – a time of relative mineralocorticoid insensitivity.^16^–^19^ Furthermore, children with cortisol deficiency may be volume depleted if unwell, or may be prone to hyponatraemia due to reduced free-water clearance, which might be interpreted as a salt-losing state in the acute clinical scenario.

Here, we describe severe loss-of-function mutations in MC2R in seven children from three kindred, several of whom had evidence of disturbed renin-sodium homeostasis and were receiving mineralocorticoid replacement. These children were originally thought to have a recessive form of primary adrenal hypoplasia. Thus, defining the molecular basis of their condition has significant implications for counselling, treatment and long-term prognosis.

## Methods

### Patients

A total of 22 individuals who had been diagnosed with likely salt-losing forms of adrenal hypoplasia were included in the study. Of these, 19 were isolated cases and 3 were familial cases. In all cases, mutations in the nuclear receptors DAX1 (NR0B1) and steroidogenic factor 1 (SF1, NR5A1) had been excluded, as well as obvious steroid biosynthetic defects, metabolic and autoimmune disorders affecting adrenal function, and adrenal haemorrhage.

### Mutational analysis of MC2R

Following Institutional Board Approval and with informed consent, genomic DNA was extracted from peripheral blood lymphocytes and the entire coding region of *MC2R* (exon 2) was amplified using variations on conditions reported previously.^3^ Polymerase chain reaction (PCR) products were purified using 1 unit/ml Exonuclease I (New England Biolabs, Hitchin, UK) and 0·1 unit/ml Shrimp Alkaline Phosphatase (USB, Cleveland, OH, USA) and then subjected to direct sequencing using BigDye terminator version 1·1 (Applied Biosystems, Foster City, CA, USA) and a MegaBACE1000™ capillary DNA sequencer (Amersham Biosciences Inc., Little Chalfont, UK). Sequence Analyser version 3·0 (Amersham Biosciences Inc.) and Sequencher version 4·1 (Genecodes Corp., Ann Arbor, MI, USA) were used to analyse the data.

### Plasma renin activity assays

Plasma renin activity (PRA) was measured using several different independently validated assays over this 15-year study period, and are expressed as pmol angiotensin I/ml/h. In brief, PRA was measured in Patient 1 by radioimmunoassay of angiotensin I using a modification of an established method.^20^ Sensitivity of the assay was 0·3 ng/ml/h, intra-assay coefficient of variation was 4·9% and interassay coefficient of variance was 6·5%. PRA was measured in Kindred 2 by the technique reported by Dillon *et al*.^21^ A commercial radioimmunoassay (RIA) (Adaltis, Casalecchio di Reno, Italy) was used to measure PRA in Kindred 3.

## Results

### Kindred I (S74I)

**Fig. 1:**
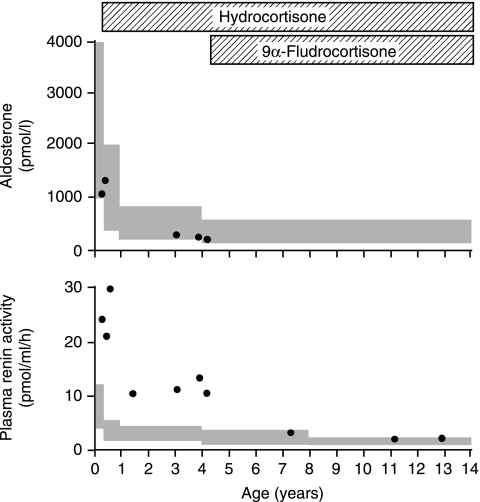
Patient 1 had low-normal aldosterone concentrations (upper panel) and persistently elevated plasma renin activity (PRA) (lower panel). The steroid replacement regimen is shown above. Shaded areas represent age-adjusted normal ranges for each assay. Recumbent measurements for PRA were taken after 2 years of age. Conversion: PRA, pmol/ml/h × 1·30 for ng/ml/h; aldosterone, pmol/l × 0·036 for ng/dl.

**Fig. 2:**
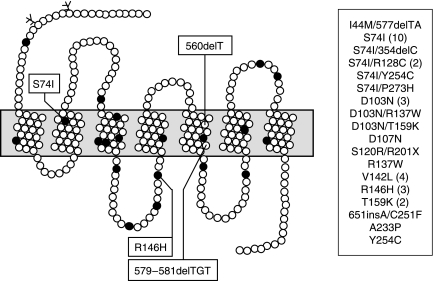
Pseudostructural plot of the ACTH receptor (MC2R) showing the position of some previously described mutations and a list of homozygous and compound heterozygous changes. Approximate number of individuals or families reported to have each individual mutation is shown in parentheses. Mutations found in the three individuals/kindred reported here (S74I; R146H/560delT; 579–581delTGT) are indicated in boxes.

Patient 1 is a female (46,XX) Causcasian child who presented with a history of prolonged jaundice, poor weight gain and progressive hyperpigmentation in the first three months of life. Initial investigations revealed elevated ACTH, hypocortisolaemia and a suboptimal cortisol response to standard synacthen stimulation (Table 1). Electrolytes were normal, but aldosterone was low for age and PRA was elevated. She was started on hydrocortisone replacement at relatively high doses (15–20 mg/m^2^/day) but had persistently elevated PRA and low aldosterone concentrations throughout early childhood (Fig. 1). Consequently, 9α-fludrocortisone treatment was started at 4 years of age, which resulted in a fall in renin activity. Mutational analysis revealed a homozygous S74I missense mutation in MC2R (Fig. 2). This mutation converts a highly conserved serine to isoleucine within the second transmembrane domain of the ACTH receptor. This change likely reduces ligand-binding affinity and signal transduction, and has been shown to be associated with marked loss of MC2R function in previous *in vitro* studies using Y6 and OS3 cells.^10^,^11^

**Table 1:** Clinical and biochemical data in patients with severe loss-of-function mutations in MC2R

Kindred Mutation	I S74I	II R146H/560delT		III 579–581delTGT			
			
Patient Sex	1 Female	1 Male	2 Female	1 Male	2 Female	3 Female	4 Female
Age at presentation	3 months	19 months	12 months	2 months	4 days	1 day	1 day
Clinical presentation	Pigmentation	Pigmentation	Pigmentation	Pigmentation	Pigmentation	Pigmentation	Pigmentation
	Hypoglycaemia	Hypoglycaemia	Hypoglycaemia	Hypoglycaemia	Hypoglycaemia	Hypoglycaemia	Hypoglycaemia
	Jaundice	Convulsion	Viral illness	Convulsion	Convulsion	Family history	Family history
ACTH (pmol/l)(normal range)	302 (< 10)	–	> 275 (< 10)	–	–	440 (< 10)	210 (< 10)
Cortisol (nmol/l) (basal/peak)	< 55/281	< 14/< 14	< 28/< 28^a^	33/10	< 14/< 14	56/45	< 14/19
PRA (pmol/ml/h)(normal range for assay/age)	24–30·3 (5·0–12)	3·2 (0·1–2·0)	1·4 (0·8–4·6)	13·5 (5·0–12)	–	–	–
Aldosterone (pmol/l)(normal range for age)	1052 (1000–4000)	–	86 (500–1500)	1189 (1000–4000)	–	–	–
Sodium (mmol/l)	137	136	129	127	147	130	138
Potassium (mmol/l)	4·3	3·9	4·0	6·5	5·7	4·3	4·6
Treatment	HC, FC	HC, FC*	HC, FC*	HC, FC	HC	HC	HC, FC

a Patient II.2 had a normal cortisol response to stimulation (peak, 769 nmol/l) at one month of age. HC, hydrocortisone; FC, fludrocortisone

* FC, fludrocortisone now withdrawn.

Conversion to SI units: ACTH, pmol/l × 4·54 for pg/ml; cortisol, nmol/l × 0·036 for µg/dl; plasma renin activity (PRA), pmol/ml/h × 1·30 for ng/ml/h; aldosterone, pmol/l × 0·036 for ng/dl.

### Kindred II (R146H/560delT)

The second family are from Iran, with an affected boy and girl. An older child had died at 18 months of age following a convulsion. The proband (46,XY) presented with failure to thrive, hyperpigmentation and a hypoglycaemic convulsion at 19 months of age in Iran. Cortisol was found to be low and he was started on methylprednisolone. At 21 months of age he was transferred for further investigation. Two days after stopping all treatment, there was no cortisol increase following standard synacthen stimulation and basal PRA was mildly elevated (Table 1). Serum 17-hydroxyprogesterone (17-OHP) was not elevated. Given these findings**,** he was diagnosed as having adrenal hypoplasia and treated with hydrocortisone and fludrocortisone throughout childhood. The fludrocortisone has recently been stopped at the age of 14 and his PRA has remained within normal limits over 18 months.

His sister's karyotype is 46,XX. She was investigated at 1 month of age on account of the family history. She had a normal cortisol response to standard synacthen stimulation at one month of age (peak 769 nmol/l), but became progressively pigmented throughout the first year of life. Investigations at 12 months of age showed absent cortisol response to synacthen stimulation and elevated ACTH. Her aldosterone was low but PRA was not raised. Almost simultaneously, she developed a severe viral illness and became hyponatraemic (sodium 129 mmol/l). She was started on hydrocortisone and received fludrocortisone treatment transiently.

Mutational analysis revealed compound heterozygous mutations (R146H/560delT) in MC2R in both children (Fig. 2). The R146H change affects a highly conserved arginine in the second intracellular loop of the receptor. This mutation has been reported previously in a homozygous state in three patients, and was carried by the mother. The arginine to histidine change likely affects the interaction between the second cytoplasmic loop and fourth transmembrane domain, resulting in severe loss of receptor function in Y6 cells through impaired G-protein coupling and ligand binding.^10^ The second change is a single nucleotide deletion at position 560 (560delT) resulting in a frameshift and premature stop codon at amino acid 215. This novel change would be predicted to cause complete loss of receptor function, and was carried by the father.

### Kindred III (579–581delTGT)

The third family is a highly consanguineous kindred from Qatar. Four of 12 children born in three nuclear families have primary adrenal failure. The proband (46,XY) presented at 60 days of age with pigmentation and a hypoglycaemic convulsion (Table 1). He had an elevated PRA, his aldosterone was inappropriately low, and he had mild hyponatraemia/hyperkalaemia. Thus, he was diagnosed as having adrenal hypoplasia and treated with hydrocortisone and fludrocortisone. The second child (46,XX) presented at 4 days of age with a hypoglycaemic convulsion. She was hyperpigmented and found to be profoundly hypocortisolaemic (< 14 nmol/l), and was started on hydrocortisone alone. Given the emerging family history, two further children were diagnosed as likely affected on the first day of life on account of marked hyperpigmentation and hypoglycaemia, and hydrocortisone was started almost immediately. One child was also commenced on fludrocortisone as it was believed this family had an autosomal recessive form of adrenal hypoplasia, and that treatment should be started before the onset of a salt-losing crisis. Thus, in all three cases, treatment was commenced within the first four days of life before disturbances in PRA or salt balance would likely have become manifest.

Mutational analysis revealed a novel homozygous three-nucleotide deletion (579–581delTGT) in all affected individuals. This deletion results in a stop codon at position 193 (Y193X) in the fifth transmembrane domain of the ACTH receptor (Fig. 2). Their parents are all carriers of this mutation and siblings are either heterozygous or normal.

## Discussion

The clinical diagnosis of adrenal failure in infancy or childhood can be challenging. Affected individuals often have an insidious clinical course, and symptoms and signs can be nonspecific or subtle. Hyperpigmentation is often overlooked, and when a child presents collapsed or with a hypoglycaemic convulsion, life-saving treatment must be administered.

When the child has marked hyponatraemia, hyperkalaemia, inappropriately high urinary sodium excretion and a reduced circulating volume, the diagnosis of salt-losing primary adrenal failure is usually clear. Such salt-losing forms of adrenal insufficiency causing combined glucocorticoid and mineralocorticoid insufficiency can occur in several well-defined conditions, such as defects in steroidogenesis (e.g. steroidogenic acute regulatory protein (STAR), CYP11A1, HSD3B2, CYP21), following adrenal destruction (e.g. haemorrhage, adrenoleukodystrophy, autoimmune), or with forms of congenital adrenal hypoplasia (e.g. X-linked (DAX1), steroidogenic factor-1, IMAGe).^22^,^23^

However, if biochemical findings are subtle (e.g. mild hyponatraemia with borderline high potassium), or if the child has already received fluid resuscitation and steroid treatment, dissecting out the exact biochemical nature of the condition at a later date can be difficult.

The association of salt-losing states or apparent mineralocorticoid insufficiency with forms of familial glucocorticoid deficiency/ACTH resistance syndromes is less clear. Spark *et al*. reported an impaired aldosterone response to ACTH stimulation in a patient with FGD, but the molecular diagnosis is not reported.^24^ Furthermore, this patient presented in childhood with isolated glucocorticoid insufficiency, no clinical disturbances in the renin-angiotensin-aldosterone pathway were ever detected, and an impairment of aldosterone response to ACTH stimulation was only evident in the recumbent setting when the individual was assessed in early adulthood. In addition, Davidai *et al*. described mild disturbances in the renin-angiotensin system in a patient with FGD.^25^ However, a MC2R mutation causing FGD1 in this individual has now been excluded (Z. Hochberg, personal communication). Thus, although it is known that approximately 10–15% of individuals who have ACTH resistance as part of the Triple A (Allgrove) syndrome (AAAS, ALADIN) (OMIM: 231550) do have evidence of mineralocorticoid deficiency,^26^ disturbances in renin-sodium homeostasis in patients with other ACTH-resistance syndromes (FGD1 due to MC2R mutations; FGD2 due to mutations in MRAP [607398]) are not well established.^27^

In normal physiological states, angiotensin II is the major drive to mineralocorticoid production. However, the ACTH receptor is expressed in the zona glomerulosa of the adrenal gland, and acute administration of synacthen to normal individuals produces a rapid and dose-dependent rise in serum aldosterone.^13^–^15^ Thus, ACTH may have an important facilitative role stimulating mineralocorticoid synthesis and release, and functional studies have shown that the CYP11B2 minimal promoter is cAMP responsive.^28^ This facilitative effect of ACTH on mineralocorticoid release may be most important during times of acute stress (e.g. illness, heat, convulsion), salt-deprivation (e.g. breastfeeding), or when there is relative mineralocorticoid insensitivity (e.g. early infancy). Consequently, one might expect ACTH resistant states to be associated with some disturbance in renin-sodium homeostasis. However, this is not well established in FGD1, as the changes described previously are often homozygous or compound heterozygous missense changes in MC2R that have some limited residual activity.^10^,^11^

The individuals described here likely represent the most severe end of the spectrum of FGD1, and include a family who are homozygous for a severely disruptive frameshift mutation in the receptor (579–581delTGT leading to a stop codon at 193). In this kindred, the proband presented with a hypoglycaemic convulsion and hyperpigmentation at 60 days of age. Subsequent affected individuals were diagnosed in the first four days of life on account of marked hyperpigmentation and hypoglycaemia, before the usual onset of salt loss or when biochemical evidence of salt loss might be manifest. Given the family history, steroid replacement was started immediately, and one child received fludrocortisone as it was felt the family had a recessive form of adrenal hypoplasia. In kindred II (R146H/560delT), the proband presented with a hypoglycaemic convulsion at 19 months of age, after an insidious period of failure to thrive. The elevated renin led to a diagnosis of adrenal hypoplasia, and it was considered necessary to add fludrocortisone treatment to the glucocorticoid replacement. Of note, his younger sister has a very adequate cortisol response to synacthen stimulation at one month of age, but was profoundly cortisol deficient by one year. She was found to be hyponatraemic when she presented with a respiratory viral illness. As her plasma renin activity was low, the low sodium could have been due to inappropriate ADH release or an inability to excrete a water load due to hypocortisolaemia. However, in the clinical context and with the family history, it was felt that she had a possible salt-losing form of adrenal hypoplasia. Finally, Patient 1 – who harbours a severe loss-of-function missense mutation (S74I) – had long-standing evidence of subtle disturbances in renin-aldosterone and was eventually commenced on fludrocortisone replacement at 4 years of age (Fig. 1) as it was felt she had a subtle form of adrenal hypoplasia. This S74I mutation is one of the more frequent mutations reported in MC2R and has been reported in approximately 10 patients previously.^2^ To our knowledge, none of these other patients have received mineralocorticoid replacement.

Taken together, these cases highlight that severe loss-of-function mutations in MC2R may be found in a significant proportion of children with primary adrenal insufficiency and who have been diagnosed as having salt losing forms of adrenal hypoplasia. These findings may suggest a supportive role for ACTH in mineralocorticoid synthesis and release, especially in times of stress, salt-restriction or relative mineralocorticoid insensitivity. In other situations, hyponatraemia may reflect inappropriate ADH release or relative water overload following infection or convulsions, especially in a glucocorticoid deficient state. As biochemical data are often limited in the acute clinical situation, and the emphasis is on saving the child's life, it is possible that many more children with sporadic or familial forms of adrenal disease have been labelled as having adrenal hypoplasia or related conditions, whereas the true pathology lies in the MC2R. This study highlights the importance of obtaining accurate data on plasma renin activity, aldosterone and salt balance in all infants and children diagnosed with primary adrenal failure, with particular attention being paid to assay- and age-specific normal ranges. Making the correct diagnosis has important implications for counselling and long-term management. Mineralocorticoid requirements often decrease with age, as evidenced from the fall in normal mineralocorticoid secretion rates after infancy and from the biochemical improvement throughout childhood seen in children with autosomal dominant pseudohypoaldosteronism type I due to mutations in the mineralocorticoid receptor. Thus, infants or young children who appear to have salt loss should be started on fludrocortisone replacement, especially if there is persistently elevated PRA, urinary sodium loss, salt craving or poor growth, or if these parameters are not normalized on a standard regimen of glucocorticoid replacement alone. However, it might be predicted that, with careful monitoring, these children could be weaned off their fludrocortisone supplements eventually, or at least to reserve them for times of stress or potential volume depletion. This situation contrasts with individuals with X-linked adrenal hypoplasia who are likely to require mineralocorticoid replacement for life. Finally, the normal cortisol response to synacthen stimulation in a one-month-old sibling, followed by profound cortisol insufficiency at one year of age, shows that FGD1 may be a clinically progressive condition, and ongoing clinical vigilance or molecular analysis are needed in relatives even if physiological tests of cortisol reserve are initially normal.
